# Assessment of knowledge, behavior and practices related to oral health and oral lesions among dental OPD patients in India

**DOI:** 10.6026/973206300222873

**Published:** 2026-05-31

**Authors:** Vaibhava Raaj, Subhash Kumar, Qalbi Fatima, Pallavi Priya, Imran Md, Abhishek Sinha

**Affiliations:** 1 Department of Dentistry, Patna Medical College & Hospital, Patna, Bihar, India

**Keywords:** Oral knowledge, oral behavior, oral issues, tobacco consumption, oral survey

## Abstract

Poor oral health knowledge and practices contribute significantly to the high burden of oral diseases, particularly in developing regions. Therefore, it is of interest to assess knowledge, behavior and practices related to oral health and oral lesions among patients attending a dental outpatient department (OPD). Hence, a cross-sectional survey of 500 adults was conducted using a structured questionnaire along with clinical oral examination. Only 38.4% demonstrated adequate knowledge and 31.6% showed positive oral hygiene behaviors, while 23.8% presented with oral mucosal lesions significantly associated with tobacco use. Thus, we show the urgent need for targeted oral health education and tobacco cessation interventions to reduce disease burden.

## Background:

The oral health is a part of the overall systemic health and quality of life and it does not only mean the absence of the disease but functional ability to speak, eat, socialize and express emotions without discomfort, pain and embarrassments [1]. It has been noted by the World Health Organization that oral diseases have been found to afflict some 3.5 billion individuals in the world, which makes oral diseases one of the most widespread non-communicable conditions in the world and dental caries in fixed teeth is the most widespread health condition affecting humanity [2]. Oral disease is disproportionately high in India and the national oral health surveys always record high prevalence of dental caries, periodontal disease, malocclusion and mouth mucosal lesions among all ages and socio-economic groups [3]. The Bihar state is one of the most problematic landscapes in terms of oral health because of its specific socio-demographic features, i.e., high population density, lower literacy rates than the national average, high levels of poverty, poor infrastructure in the health sector in rural regions and firmly established practice of consuming tobacco and betel nuts that predisposes population to a range of oral diseases including potentially malignant ones [4]. Even with these identified risk factors, there is a very large gap in the systematic data on oral health literacy and behavioral patterns as well as disease prevalence among the population seeking dental care in Bihar, which will result in an evidence gap that prevents the development of effective approaches to public health. Knowledge, Behavior and Practice surveys conceptual frameworks have been well utilized in the research of the public health as a standardized research methodology of measuring the cognitive, attitudinal and behavioral determinants of health outcomes of specific populations [5]. Knowledge in the context of oral health include knowledge of disease etiology, disease prevention and knowledge of pathological conditions; behavior refers to the behavioral patterns of oral hygiene, dietary behavior and substance use patterns, directly related to the health of the oral tissues; practice refers to health-seeking patterns of oral health, use of dental services and self-care practices adopted by people [6]. The relationship between these three dimensions is complex and two-way knowledge does not ensure positive behavior and intentions to behave are not always converted into a regular practice [7]. Oral mucosal lesions present a very significant aspect of oral health examination, which is not often included in the community survey, which mainly covers the issue of dental caries and periodontal disease. The country of India is facing one of the largest oral cancer loads in the world, it is estimated that 1/3rd of all oral cancer cases in the world are in India with age standardized incidences rates far beyond those of the Western world [8]. Most of oral cancers are preceded by the clinically identifiable potentially malignant disorders such as leukoplakia, erythroplakia, oral submucous fibrosis and oral lichen planus that offer a window of opportunity to detect and intervene at an early stage that can significantly lower the morbidity and mortality rates [9]. The awareness of these precancerous conditions among patients themselves and among healthcare providers during the regular checkup is thus of utmost importance to the general health of the people. The relationship between the prevalence of oral mucosal lesions and the use of tobacco in its different varieties has been widely established. The shape of tobacco use is unique in the Indian context that requires not only smoking cigarettes but also rich repertoire of smokeless tobacco products such as gutka, khaini, zarda and betel quid preparations, which involves carcinogenic substances in direct contact with the oral mucosa over an extended period of time [10, 11]. Cultural acceptance, low price, easy access and poor knowledge on health hazards have made smokeless tobacco use one of the most common in Bihar, India. Knowledge, attitudes and practices of the dental outpatient population with regard to tobacco use are thus critical in the formulation of the effective cessation counseling programs. Past oral health surveys in different states in India have established high levels of knowledge gaps and adverse oral health behavior among different population groups. Research in urban and semi-urban areas has indicated that a significant percentage among adults are using not toothbrushes but traditional means of cleaning their teeth and have little knowledge about the etiology and prevention of the most frequent oral diseases and that they use dental services not as a preventive measure but as a palliative one [12]. Nevertheless, specific knowledge, behavior and practice profile of patients visiting governmental dental clinics in Bihar have not been systematically defined yet, which is a population that might not be very similar to those investigated in other Indian states because of the differences in the local sociocultural and economic background [13]. In addition to this, the majority of the current surveys have determined the knowledge and oral health behavior, but have not conducted a clinical examination at the same to compare self-reported practices with the real oral health and disease condition [14]. Therefore, it is of interest to evaluate knowledge, behavior and practices related to oral health and their association with the prevalence of oral lesions among dental outpatients.

## Materials and Methods:

## Study design and setting:

This cross-sectional descriptive study was conducted at the outpatient department (OPD) of the Department of Dentistry, Patna Medical College Hospital (PMCH), Patna, Bihar, India, over a period of four months from November 2025 to March 2026. Patna Medical College Hospital is a major tertiary care government teaching hospital serving a large catchment area encompassing urban Patna as well as surrounding semi- urban and rural districts of Bihar. Written informed consent was obtained from all participants in Hindi.

## Sample size calculation:

The sample size was calculated using the formula n = Z^2^pq/d^2^, where Z = 1.96 (at 95% confidence level), p = estimated prevalence of adequate oral health knowledge (assumed as 35% based on available Indian literature), q = 1 - p = 0.65 and d = desired precision (margin of error = 4.5%). The minimum calculated sample size was 456. Accounting for approximately 10% incomplete responses, 520 patients were initially approached, of whom 500 provided complete data and were included in the final analysis.

## Participant selection:

Patients attending the dental OPD during the study period were recruited through consecutive sampling.

## Inclusion criteria:

[1] Age 18 years and above

[2] Willingness to participate and provide written informed consent

[3] Patients visiting the dental OPD for any reason (new or follow-up visits)

[4] Ability to comprehend and respond to the questionnaire in Hindi or English 

## Exclusion criteria:

[1] Patients below 18 years of age

[2] Patients with severe systemic conditions precluding clinical examination

[3] Patients with cognitive impairment or communication disabilities preventing questionnaire completion

[4] Patients undergoing active treatment for oral malignancy

[5] Patients who had participated in any oral health education program within the preceding three months

## Data collection tool:

A structured, pre-tested questionnaire was developed based on previously validated oral health survey instruments adapted to the local sociocultural context. The questionnaire was initially drafted in English, translated into Hindi by two independent bilingual translators and back-translated to verify conceptual accuracy. Content validity was established through review by a panel of four experts (two oral medicine specialists, one community dentist and one public health specialist), achieving a content validity index of 0.86. The questionnaire was pilot tested among 50 patients (not included in the main study) to assess comprehension, completion time and reliability. Cronbach's alpha for the overall instrument was 0.81. Average completion time was 12 to 15 minutes.

The questionnaire comprised five sections totaling 42 items:

[1] Section I - Sociodemographic Profile (8 items): Age, sex, educational qualification, occupation, monthly family income, residential background (urban/semi-urban/rural), marital status and family size.

[2] Section II - Oral Health Knowledge (12 items): Questions assessing knowledge of causes of dental caries, gum disease etiology, role of fluoride, importance of regular dental visits, knowledge of oral cancer risk factors, awareness of oral mucosal lesions as warning signs and understanding of the link between oral and systemic health. Each correct response scored one point (maximum 12). Scores were categorized: adequate (≥ 9), moderate (5-8) and poor (≤ 4).

[3] Section III - Oral Hygiene Behavior (8 items): Tooth-cleaning method (toothbrush/finger/neem stick/other), type of dentifrice, frequency of brushing, method of brushing, tongue cleaning practice, use of interdental aids, frequency of toothbrush replacement and mouthwash use.

[4] Section IV - Dietary and Substance Use Practices (8 items): Frequency of sugar-containing food and beverage consumption, consumption of fresh fruits and vegetables, tobacco use (type, form, frequency, and duration), betel quid chewing habit and alcohol consumption.

[5] Section V - Dental Visit Behavior and Self-Perception (6 items): Reason for current dental visit (pain/routine/referral), previous dental visit history, perceived oral health status, history of noticing any oral mucosal change, action taken upon noticing oral changes and barriers to seeking dental care.

## Clinical oral examination:

Following questionnaire administration, a comprehensive clinical oral examination was performed by two calibrated examiners (inter- examiner kappa = 0.87 for oral lesion diagnosis, 0.91 for DMFT assessment) under standardized conditions using a dental chair with overhead light, mouth mirror, explorer and periodontal probe (WHO probe).

## The following clinical assessments were performed:

[1] DMFT Index: The decayed, missing and filled teeth index was recorded according to WHO criteria for all permanent teeth.

[2] Community Periodontal Index (CPI): Assessed using index teeth and WHO periodontal probe. Scores: 0 = healthy, 1 = bleeding on probing, 2 = calculus detected, 3 = shallow pocket (4-5 mm), 4 = deep pocket (≥ 6 mm).

[3] Oral Mucosal Examination: Systematic examination of all oral mucosal surfaces (labial mucosa, buccal mucosa, tongue dorsum and ventral surface, floor of mouth, hard and soft palate, gingiva, oropharynx) was performed under adequate illumination with retraction using mouth mirrors. Oral mucosal lesions were diagnosed clinically based on established diagnostic criteria and classified according to WHO classification. Lesions identified included leukoplakia, erythroplakia, oral submucous fibrosis (OSMF), oral lichen planus, aphthous ulcers, candidiasis, traumatic ulcers, tobacco pouch keratosis and other lesions. Patients with clinically suspicious lesions were referred for biopsy and histopathological confirmation.

## Statistical analysis:

Data were entered into Microsoft Excel and analyzed using SPSS version 25.0 (IBM Corp., Armonk, NY, USA). Descriptive statistics were expressed as frequencies, percentages and mean ± standard deviation. Chi-square tests were used for associations between categorical variables. Independent samples t-tests and one-way ANOVA were used for comparing continuous variables across groups. Binary logistic regression analysis was performed to identify independent predictors of oral lesion presence. Spearman's rank correlation was used to assess relationships between knowledge scores and clinical parameters. Statistical significance was set at p < 0.05.

## Results:

A total of 500 participants completed the study. The mean age was 36.8 ± 12.4 years, with an age range of 18-72 years. Males constituted 284 participants (56.8%), while females accounted for 216 participants (43.2%). Adequate oral health knowledge was observed in 192 participants (38.4%), moderate knowledge in 196 participants (39.2%) and poor knowledge in 112 participants (22.4%). The overall mean knowledge score was 6.84 ± 2.76. Adequate knowledge was significantly higher among females than males (43.5% vs 34.5%, p = 0.023). Knowledge level also showed significant association with age, education, residential background and monthly family income (p <0.05), with higher knowledge observed among younger participants, graduates, urban residents and participants with higher income, as shown in Table 1 Oral hygiene practices are presented in Table 2 Toothbrush with toothpaste was the most commonly used cleaning method, reported by 348 participants (69.6%). However, only 112 participants (22.4%) brushed twice daily or more, while 326 participants (65.2%) brushed once daily. Tongue cleaning was practiced by 268 participants ( 53.6%), whereas interdental aid use was very low, reported by only 46 participants (9.2%). Timely toothbrush replacement within three months was observed in 124 participants (24.8%). Overall favorable oral hygiene behavior, defined as brushing twice daily with toothbrush and toothpaste, regular tongue cleaning and timely toothbrush replacement, was present in only 158 participants (31.6%). Logistic regression analysis identified smokeless tobacco use as the strongest predictor of oral lesions, with an odds ratio of 4.86 (95% CI: 2.94-8.04). Tobacco duration of more than 10 years was also a significant predictor, with an odds ratio of 3.21 (95% CI: 1.87-5.51). Poor oral health knowledge increased the odds of oral lesions by 2.14 times (95% CI: 1.28-3.58), while age above 45 years, male sex and rural residence also showed increased odds (Table 3). Alcohol consumption showed a mild increase in risk but was not as strong as tobacco-related predictors. Overall, the results indicate that poor knowledge, unfavorable oral hygiene behavior and tobacco use were important factors associated with oral disease burden among patients visiting the dental outpatient department.

## Discussion:

The current research gives a thorough evaluation of oral health knowledge, behavior and practices in dental outpatients of Patna Medical College Hospital and identifies meaningful relations between oral health knowledge, behavior and practice and clinical oral health outcomes in terms of dental caries experience, periodontal status and the prevalence of oral mucosal lesions. The results indicate that all three areas, namely knowledge, behavior and practice, are highly deficient in this group of people as the rates of tobacco use and related potentially malignant oral lesions are alarming. The result that only 38.4% of the participants showed sufficient oral health knowledge is in tandem with the surveys carried out in the other regions in India but is also a worrying fact about a population that actively pursues dental services. Past state-wide surveys have cited sufficient levels of oral health knowledge between 25% and 45% with much fluctuation that is attributable to regional education, economic and cultural elements [15]. The high positive correlation between educational level and the score of knowledge in this study proves the proven relationship between formal education and health literacy and supports the value of oral health education programs at schools as a long-term measure to promote oral health awareness in the population [16]. The preponderance of pain-based dental visits (59.2%), the observation that one-quarter of the respondents were at a dental facility the first time highlight the reactive nature of dental healthcare-seeking behavior among this population group. The trend has been observed steadily across the communities in India and is attributable to a multi- faceted combination of issues such as financial limitation, lack of knowledge about preventative dental services, geographic obstacles to dental services, fear and anxiety about dental services and prioritization of curative rather than preventative healthcare in the overall Indian healthcare culture [17]. Of particular concern is the fact that the rate of periodontal dental check-ups (10.4%) is extremely low, which restricts the ability to detect dental diseases, oral mucosal lesions and possibly malignant conditions at stages when they can be treated best and the least invasive way. The behavior pattern in oral hygiene as seen in the current research brings both indications of improvement and continued issues. Although most participants (69.6) brushes their teeth with toothpaste- which is a positive increase over the older surveys that registered higher percentages of older oral cleaning methods- the frequent occurrence of brushing once a day (22.4) and the usage of interdental cleaning products (9.2) is an indication that oral cleaning habits are still at an inferior level as compared to optimal levels [18]. The fact that a percentage of 16.8% of participants still use traditional methods of cleaning their fingers (neem sticks), cleaning their teeth with charcoal and cleaning their teeth with fingers indicates that the traditional methods of cleaning their teeth have not been fully abandoned, but still, they hold great value in terms of culture, which, however, do not offer equal mechanical ability to clean their teeth in the same way that modern methods using toothbrushes [19]. The prevalence of tobacco use among the study participants of 44.8% is significantly greater than the national prevalence of the same of 44.8 in the Global Adult Tobacco Survey of India, as well as the prevalence of high tobacco consumption in Bihar as reported in the literature. The dominance of smokeless tobacco (29.2% exclusive use smokeless plus 6.0% dual use) over smoking tobacco is also in line with the unique tobacco use pattern in this area where a culturally instilled behaviour of gutka chewing, khaini use and paan intake lead to a continuous direct contact between the mucosa and carcinogenic substances [20]. This observation has direct implication in the development of oral lesions since clinical evidence of this study indicated that there is evident dose-response relationship between smokeless tobacco use, years of use and occurrence of oral lesion. The general occurrence of the oral mucosal lesions at 23.8 percent and the occurrence of possibly malignant disorders in 17.4 percent of the study group are findings that have significant implications to the overall population health. The fact that leukoplakia (7.6%) and oral submucous fibrosis (6.8%) are the most common types of lesions indicates the etiological role of the common smokeless tobacco and betel nut use among the population. These prevalence rates are higher than in general population surveys of other Indian states which have recorded oral mucosal lesion prevalence rates of 5% to 15% which perhaps reflects not only the increased rate of tobacco use in Bihar but also the selection bias in a hospital-based sample which may contain lesions that are symptomatic, prone to present themselves to the hospital [21]. The close independence relationship between smokeless tobacco use and the presence of oral lesions (OR = 4.86) by logistic regression is in line with known carcinogenic responses of smokeless tobacco products, which harbor tobacco-specific nitrosamines, polycyclic aromatic hydrocarbons and reactive oxygen species that cause genetic mutations, epigenetic changes and chronic inflammatory events in the oral mucosa [22]. The strong dose-response correlation with the duration of use also supports the causal interpretation - in users having a more than ten-year history of tobacco exposure, the prevalence of the lesions was more than 58 percent, approximately threefold the prevalence of the lesions among users with a less than five-year history of tobacco exposure. The result that the knowledge of oral health was the only independent predictor of the presence of oral lesions (OR = 2.14) despite the effects of tobacco use and demographic factors indicated that knowledge gaps are the cause of oral lesion development independent of tobacco use. Poorly informed patients can be less able to perceive the initial mucosal alterations as signs of trouble, less willing to adopt harmful behaviors, less willing to seek prompt medical care on persistent oral alterations and more willing to practice other risk behaviors such as poor oral hygiene and unhealthy dietary habits that facilitate permissive conditions to develop disease [23]. The very low percentage of patients that observed oral mucosal changes and accessed treatment (38.3) points at a severe disjunction in the secondary prevention pathway. Although patients may develop awareness of abnormal changes in their oral cavity, cultural fatalism about disease, deficiency of knowledge about the malignant potential, financial constraints to seeking health care, an asymptomatic nature of most lesions which display early potential malignancy all hinder help-seeking response in time [24]. The discovery highlights the necessity of the popularization of awareness campaigns that would make the population aware of specifics of the warning signs of oral cancer and the role of early assessment of the ongoing changes in the mouth. The large mean score of DMFT (5.84) which has a highly skewed distribution of the decayed component (3.96) and minimal filled component (0.46) means that there exist high levels of unmet restorative treatment in this group. The low filled-to-decayed ratio (1:8.6) is typical of a healthcare system with low access to dental care and with patients largely presenting with advanced disease with which it is necessary to extract instead of providing restorative care [25]. There are a number of limitations one must admit. The convenience sampling, which is based on the hospital setting, might not reflect the population of Bihar as a whole since patients seeking dental services might possess diverse knowledge, behavior and disease characteristics than those who do not seek medical services. Behavioral data that are self-reported can easily be influenced by social desirability, especially in the aspects of smoking and oral care habits. The cross-sectional design does not allow any causal inferences. Clinical diagnosis of oral mucosal lesions without histopathological confirmation of all lesions can bring about diagnostic ambiguity even though biopsy referral was made on suspicious lesions [26].

## Conclusion:

Dental outpatients showed inadequate oral health knowledge and suboptimal hygiene practices. Care largely sought for symptomatic rather than preventive reasons. High prevalence of tobacco use was strongly associated with increased occurrence of oral mucosal lesions, including potentially malignant disorders. Thus, we show the need for integrated oral health education, tobacco cessation programs and community-based preventive strategies.

## Figures and Tables

**Table 1:** Socio-demographic characteristics and oral health knowledge levels (n = 500)

**Characteristic**	**n (%)**	**Knowledge Level -Adequate n (%)**	**p-value**
**Sex**			
Male	284 (56.8%)	98 (34.5%)	0.023
Female	216 (43.2%)	94 (43.5%)	
**Age Group**			
18-30 years	178 (35.6%)	82 (46.1%)	
31-45 years	168 (33.6%)	64 (38.1%)	0.004
46-60 years	108 (21.6%)	34 (31.5%)	
> 60 years	46 (9.2%)	12 (26.1%)	
**Education Level**			
Illiterate	72 (14.4%)	8 (11.1%)	
Primary school	96 (19.2%)	22 (22.9%)	
Secondary school	124 (24.8%)	46 (37.1%)	< 0.001
Higher secondary	104 (20.8%)	52 (50.0%)	
Graduate and above	104 (20.8%)	64 (61.5%)	
**Residential Background**			
Urban	196 (39.2%)	98 (50.0%)	
Semi-urban	148 (29.6%)	54 (36.5%)	< 0.001
Rural	156 (31.2%)	40 (25.6%)	
**Monthly Family Income**			
< ₹10,000	164 (32.8%)	38 (23.2%)	
₹10,000-25,000	188 (37.6%)	72 (38.3%)	< 0.001
> ₹25,000	148 (29.6%)	82 (55.4%)	
**Overall Knowledge Level**			
Adequate (≥ 9)	192 (38.4%)	-	-
Moderate (5-8)	196 (39.2%)	-	-
Poor (≤4)	112 (22.4%)	-	-
Knowledge Score, Mean ± SD	6.84 ± 2.76	-	-

**Table 2:** Oral health behaviors, substance use practices and dental visit patterns (n = 500)

**Parameter**	**n (%)**
**Tooth Cleaning Method**	
Toothbrush with toothpaste	348 (69.6%)
Toothbrush with tooth powder	68 (13.6%)
Finger with toothpaste/powder	42 (8.4%)
Neem stick/datun	32 (6.4%)
Charcoal/ash/other traditional	10 (2.0%)
**Brushing Frequency**	
Twice daily or more	112 (22.4%)
Once daily	326 (65.2%)
Irregular/less than daily	62 (12.4%)
Tongue Cleaning Practice	268 (53.6%)
Use of Interdental Aids (floss/brush)	46 (9.2%)
Frequency of Toothbrush Replacement	
Every 3 months or sooner	124 (24.8%)
Every 3-6 months	186 (37.2%)
Every 6-12 months	118 (23.6%)
More than 12 months/when frayed	72 (14.4%)
Sugar-Containing Food/Beverage (≥3 times/day)	186 (37.2%)
Tobacco Use (Any Form)	224 (44.8%)
Smokeless tobacco only	146 (29.2%)
Smoking tobacco only	48 (9.6%)
Both forms	30 (6.0%)
**Types of Smokeless Tobacco Used**	
Gutka	82 (16.4%)
Khaini	64 (12.8%)
Paan with tobacco	48 (9.6%)
Zarda/other	32 (6.4%)
Duration of Tobacco Use (among users)	
< 5 years	68 (30.4%)
5-10 years	84 (37.5%)
> 10 years	72 (32.1%)
Betel Nut (Supari) Chewing (without tobacco)	88 (17.6%)
Alcohol Consumption (regular)	76 (15.2%)
**Reason for Current Dental Visit**	
Pain/emergency	296 (59.2%)
Routine check-up	52 (10.4%)
Specific complaint (swelling/bleeding/mobility)	108 (21.6%)
Referral from other department	44 (8.8%)
**Previous Dental Visit History**	
First-ever dental visit	128 (25.6%)
Visited > 1 year ago	204 (40.8%)
Visited within past year	168 (33.6%)
**Self-Perceived Oral Health**	
Good	124 (24.8%)
Average	228 (45.6%)
Poor	148 (29.6%)
Noticed Any Oral Mucosal Change	94 (18.8%)
Sought Treatment for Mucosal Change	36 (38.3% of those who noticed)

**Table 3:** Clinical findings and associations with knowledge and tobacco use

**Panel A: Clinical Parameters**	**Mean ± SD or n (%)**
DMFT Score, Mean ± SD	5.84 ± 3.72
Decayed component (D)	3.96 ± 2.81
Missing component (M)	1.42 ± 1.68
Filled component (F)	0.46 ± 0.92
CPI - Healthy (Score 0), n (%)	62 (12.4%)
CPI - Bleeding (Score 1), n (%)	98 (19.6%)
CPI - Calculus (Score 2), n (%)	196 (39.2%)
CPI - Shallow Pocket (Score 3), n (%)	108 (21.6%)
CPI - Deep Pocket (Score 4), n (%)	36 (7.2%)
Oral Mucosal Lesions - Overall Prevalence	119 (23.8%)
Leukoplakia	38 (7.6%)
Oral submucous fibrosis	34 (6.8%)
Oral lichen planus	14 (2.8%)
Aphthous ulcers	12 (2.4%)
Tobacco pouch keratosis	11 (2.2%)
Candidiasis	6 (1.2%)
Traumatic ulcer	4 (0.8%)
**Panel B: Oral Lesion Prevalence by Subgroup**	**Lesion Present n (%)**
Tobacco Users (n = 224)	89 (39.7%)
Non-Tobacco Users (n = 276)	30 (10.9%)
Smokeless Tobacco Users (n = 176)	78 (44.3%)
Smokers Only (n = 48)	11 (22.9%)
Duration > 10 years (n = 72)	42 (58.3%)
Duration < 5 years (n = 68)	14 (20.6%)
Poor Knowledge (n = 112)	42 (37.5%)
Adequate Knowledge (n = 192)	28 (14.6%)
**Panel C: Logistic Regression - Predictors of Oral Lesions**	**OR (95% CI)**
Smokeless tobacco use	4.86 (2.94-8.04)
Tobacco duration > 10 years	3.21 (1.87-5.51)
Poor oral health knowledge	2.14 (1.28-3.58)
Age > 45 years	1.82 (1.12-2.96)
Male sex	1.67 (1.04-2.69)
Rural residence	1.48 (0.92-2.38)
Alcohol consumption	1.39 (0.81-2.38)
